# The impact of trait mindfulness on work engagement among primary and secondary school teachers: a moderated mediation model

**DOI:** 10.3389/fpsyg.2025.1640996

**Published:** 2025-08-13

**Authors:** Bo Han, Kai Zhang

**Affiliations:** ^1^School of Humanities, Southeast University, Nanjing, China; ^2^School of Education, Huaibei Normal University, Huaibei, China

**Keywords:** trait mindfulness, work engagement, job satisfaction, perceived organizational support, teachers

## Abstract

Work engagement not only improves teachers’ job performance but also contributes to enhancing their students’ academic outcomes and thereby promoting the latter’s physical and mental development. Teacher mindfulness can affect work engagement. To clarify the relationship between trait mindfulness and work engagement and its underlying mechanisms, this study constructed a moderated mediation model based on the Conservation of Resources Theory (COR), specifically examining the mediating role of job satisfaction and the moderating role of perceived organizational support. A questionnaire survey was conducted among 3,225 primary and secondary school teachers using convenience sampling, and data were processed with SPSS and Mplus statistical software to assess their states of trait mindfulness, work engagement, job satisfaction, and perceived organizational support. The results showed: (1) Trait mindfulness positively significantly predicted work engagement; (2) Job satisfaction positively mediated the relationship between trait mindfulness and work engagement; (3) Perceived organizational support positively moderated the relationship between trait mindfulness and work engagement; (4) Perceived organizational support also positively moderated the relationship between trait mindfulness and job satisfaction, as well as the mediating effect of job satisfaction between trait mindfulness and work engagement. The findings deepen the theoretical understanding of the interaction mechanisms between trait mindfulness and work engagement and offer practical insights into enhancing teachers’ work engagement through trait mindfulness.

## 1 Introduction

Work engagement has significant positive implications for teachers, their students, and their schools (Lipscomb et al., 2022; van Roekel et al., 2024; Uslukaya and Zincirli, 2025). High levels of work engagement benefit teachers’ professional development (Lyons, 2006), enhance their psychological resilience and well-being (Rusu and Colomeischi, 2020), and improve self-efficacy, job satisfaction, social support, and creativity (Derakhshan et al., 2023; Fathi et al., 2024; Perera et al., 2018). High work engagement also leads to more effective teaching, improved student academic performance, and positive social behavior (Soininen et al., 2023), positively impacting teaching outcome and students’ development (Li, 2019). Teachers with low work engagement often experience burnout and other mental health issues (Martínez et al., 2024).

Work engagement as a critical solution to teacher turnover and burnout (Martínez et al., 2019). Due to the significant work pressure that teachers face (Skaalvik and Skaalvik, 2014), an increasing number of them are exhibiting symptoms of burnout (Li, 2019; Martínez et al., 2024). With the rise of positive psychology, studies on teacher burnout have increasingly turned to teacher work engagement (Bakker and Albrecht, 2018). Mindfulness is an important way to regulate teacher work engagement (Coo and Salanova, 2018; Leroy et al., 2013). Existing evidence suggests that mindfulness is a key psychological resource for maintaining teachers’ occupational health, positively affecting their vitality and focus (Frank et al., 2016). It has a clear influence on teacher work engagement (Tao, 2022). Teacher mindfulness can directly affect work engagement or indirectly influence it through mediators such as positive emotions, emotional intelligence, and psychological resilience (Tao, 2022).

Moreover, the current literature mainly centers on the pairwise relationships among variables such as mindfulness and work engagement, mindfulness and job satisfaction, as well as job satisfaction and work engagement, while neglecting the research on the complex interrelationships among multiple relevant variables. According to the Conservation of Resources Theory (COR), the root of pressure lies in the loss or threat of resources, while effective coping depends on maintaining resource equilibrium, preventing resource depletion, and facilitating resource accumulation (Hobfoll, 1989). Mindfulness, perceived organizational support, and job satisfaction—representing the dimensions of intrinsic personal capacity, external social support, and positive affective states, respectively provide teachers with resources to mitigate pressure, reduce burnout, and enhance work engagement (Schaufeli and Bakker, 2004; Dane and Brummel, 2014; Eisenberger et al., 1986; Hülsheger et al., 2013; Judge and Kammeyer-Mueller, 2012; Kurtessis et al., 2017; Rhoades and Eisenberger, 2002). Consequently, this study selects trait mindfulness, work engagement, job satisfaction, and perceived organizational support as the core research variables to examine the mediating and moderating effects of relevant variables, identify the mechanisms underlying their synergistic operation, and enrich the theoretical research on the relationship between mindfulness and work engagement.

Despite the considerable effect of mindfulness on work engagement documented in the available literature, research on the relationship between the two constructs in the context of Chinese teachers remains limited (Li, 2015; Liu et al., 2020; Tao, 2022). Given the largest educator workforce of the Chinese teachers, ranking top worldwide (Wu et al., 2025), studying the mechanisms in Chinese context, as was with this present study, is of great significance (Li, 2019; Martínez et al., 2024; Tao, 2022). Thus, this study will also offer valuable insights for educational administrators in policy-making and for teachers’ self-regulation.

## 2 Literature review and theoretical hypotheses

### 2.1 Trait mindfulness and work engagement

#### 2.1.1 Mindfulness and trait mindfulness

Mindfulness is defined as an observational, non-judgmental stance of the subconscious (Bishop et al., 2004; Brown and Ryan, 2003; Brown et al., 2007). Mindfulness emphasizes openness and acceptance, experiencing and accepting one’s thoughts and feelings with awareness and non-judgment (Kabat-Zinn, 1990, 1994). Growing evidence suggests that mindfulness is negatively correlated with psychological issues such as depression, anxiety, and pressure (Keleynikov et al., 2022). Due to its positive effects on mental health, burnout, and interpersonal relationships, mindfulness has become an important concept in psychology and education (Fathi et al., 2023; Wang, 2023). Individuals with high mindfulness are typically perceived as having stronger cognitive and self-regulatory abilities (Zhu, 2022). Thus, mindfulness significantly improves teachers’ burnout, mental health, and teaching quality (Flook et al., 2013), helping them manage emotions and focus on classroom challenges (Mendelson et al., 2023; Pan and Liu, 2022). In short, mindfulness plays a vital role in enhancing teachers’ mental health and quality of life (Song and He, 2021).

As mindfulness research has evolved, it has become a multifaceted concept (Duan, 2014). Mindfulness can refer to a psychological process (Bishop et al., 2004), a state of present-moment awareness with a conscious and non-judgmental stance (“state mindfulness”) (Brown and Ryan, 2003), a specific attention-training intervention (Baer, 2003), or a relatively stable personality trait (“trait mindfulness”) (Baer et al., 2006; Duan, 2014; Yang, 2021). Trait mindfulness can be formed as a habitual pattern through the repeated activation of state mindfulness via meditation practice (Goleman and Davidson, 2017; Kiken et al., 2015). The cultivation of mindfulness practice and the cumulative effects of state mindfulness engagement can enhance trait mindfulness (Kiken et al., 2015; Warren et al., 2023). Therefore, research on individual differences in mindfulness has largely focused on trait mindfulness (Glomb et al., 2011), which has become a common dimension in mindfulness studies (Kiken et al., 2015; Yang, 2021).

Trait mindfulness reflects an individual’s tendency and ability to maintain mindfulness in daily life, making the construct a relatively stable trait reflecting one’s mindfulness (Brown and Ryan, 2003; Kiken et al., 2015). Additionally, as a trait-like variable (Duan, 2014; Hülsheger et al., 2013), trait mindfulness represents cross-situational, stable individual differences (Brown et al., 2007; Song et al., 2021). Moreover, according to afore-mentioned COR theory, and Job Demands-Resources (JD-R) theory, the stability of trait mindfulness establishes it as a crucial psychological resource for individuals. Furthermore, given the long-term and sustained nature of employees’ professional behaviors, which require mindfulness to function consistently, trait mindfulness has become the fundamental perspective for assessing employee mindfulness (Mesmer-Magnus et al., 2017; Xiao, 2024). Due to the stability of trait mindfulness and its applicability in evaluating employees’ mindfulness, in our investigation of Chinese teachers’ mindfulness, we set our special focus on their trait mindfulness, conceptualizing it as a construct that reflects the teachers’ mindfulness capacity and proficiency level. Accordingly, we used the “trait mindfulness” and “mindfulness” interchangeably throughout.

#### 2.1.2 Work engagement

Work engagement refers to a positive, fulfilling work-related state characterized by vigor, dedication, and absorption (Bakker and Demerouti, 2008; Schaufeli and Bakker, 2010; Schaufeli et al., 2006; Schaufeli et al., 2002; Wang and Pan, 2023). Engaged individuals exhibit high energy, enthusiasm, and focus, actively integrating physically, cognitively, and emotionally into their work (Bakker and Demerouti, 2008; Hu and Wang, 2014). From the perspective of positive psychology, factors influencing work engagement include social support, professional self-efficacy, positive emotions, sleep quality, and core self-evaluations. Work engagement enhances job satisfaction, performance, well-being, proactive learning behaviors, and positive psychological capital (Hu and Wang, 2014). Thus, improving work engagement is a key means to enhance work effectiveness (Lv and Hu, 2023). Teacher work engagement exhibits similar characteristics. Based on Schaufeli et al.’s definition, teacher work engagement refers to their proactive state and identification with educational activities (Schaufeli and Bakker, 2010; Schaufeli et al., 2002; Wang and Tian, 2024). Teacher work engagement is closely linked to self-efficacy, social support, job satisfaction, and creativity (Derakhshan et al., 2023). Work engagement is considered a critical competency for teachers (Liu et al., 2023; Perera et al., 2018; Schaufeli et al., 2006). High teacher engagement not only signifies high self-efficacy and well-being (Rusu and Colomeischi, 2020) but also fosters positive teacher-student interactions (Zhang and Yang, 2021) and reflects a positive psychological state and effective work outcomes (Fathi et al., 2024).

#### 2.1.3 Trait mindfulness and work engagement

In light of the COR theory, mindfulness is a positive psychological resource that enhances work engagement (Good et al., 2016). Mindfulness positively influences work engagement; higher mindfulness levels correlate with greater work engagement (Leroy et al., 2013; Liu et al., 2020; Zhi and Wang, 2025). Mindfulness affects work engagement through three pathways: attention stability, self-awareness, and self-regulation (Brown et al., 2007; Glomb et al., 2011). Mindfulness helps teachers focus and enhances self-awareness, thereby improving work effectiveness. Existing research confirms a positive correlation between teacher mindfulness and work engagement (Tao, 2022). However, our understanding of the mechanisms linking trait mindfulness and work engagement remains limited.

Thus, the following hypothesis can be proposed:

*H1*: Trait mindfulness is positively correlated with work engagement among Chinese primary and secondary school teachers.

### 2.2 Trait mindfulness, job satisfaction, and work engagement

#### 2.2.1 Trait mindfulness and job satisfaction

Job satisfaction refers to the positive or negative emotional experiences individuals have in work settings (Davis et al., 1989; Locke, 1969). Some views define job satisfaction as an evaluation or judgment of one’s work situation (Weiss, 2002), considering it a cognitive assessment of emotional states rather than an emotional response (Rayton and Yalabik, 2014). Research shows that mindfulness influences attention, shaping cognition, emotions, behavior, and physiology (Good et al., 2016). Among these, cognition and emotions are the most closely related pathways through which mindfulness affects work and life (Hockey, 2000). A widely accepted definition of job satisfaction is “a pleasurable or positive emotional state resulting from the appraisal of one’s job” (Locke, 1976). Overall, job satisfaction encompasses both cognitive evaluations of work-related emotions and emotions based on work cognition (Hulin and Judge, 2003). Thus, teacher job satisfaction can be described as the positive or negative emotions teachers experience in school settings, reflecting their multifaceted affective experiences, attitudes, and evaluations (Yin et al., 2024). Studies indicate that teacher job satisfaction affects their enthusiasm, school education quality, and mental health (Burić and Moè, 2020; Toropova et al., 2021).

Trait mindfulness is positively correlated with job satisfaction (Mesmer-Magnus et al., 2017). Self-Determination Theory posits that trait mindfulness may positively correlate with job satisfaction through self-determined behavior (Glomb et al., 2011), as mindfulness promotes actions aligned with personal needs and values (Brown and Ryan, 2003). Existing research supports a positive correlation between trait mindfulness and job satisfaction (Brown and Ryan, 2003; Hülsheger et al., 2013). Employees who undergo mindfulness training show significant improvements in job satisfaction (Pang and Ruch, 2019). Therefore, mindfulness can enhance teacher job satisfaction (Barata-Gonçalves et al., 2024). Mindfulness training for teachers significantly improves their health, well-being, and job satisfaction (Roeser et al., 2012). Studies on preschool teachers indicate that trait mindfulness can predicts their job satisfaction (Song et al., 2021).

#### 2.2.2 Job satisfaction and work engagement

Job satisfaction and work engagement are positively correlated (Aziz et al., 2021; Bocean et al., 2020), with a bidirectional relationship. Extensive evidence suggests that job satisfaction is an outcome of work engagement (Sahito and Vaisanen, 2020; Yalabik et al., 2013). Work engagement is intrinsic to the job itself (Maslach and Leiter, 1997). Thus, work engagement differs from job satisfaction; engagement reflects a proactive psychological and work state, whereas job satisfaction is a more passive form of employee well-being (Bakker, 2011). Work engagement precedes job satisfaction (Cole et al., 2012; Sharma and Sood, 2023). Highly engaged individuals tend to be more satisfied with their work. However, many studies argue that job satisfaction significantly predicts work engagement (Høigaard et al., 2012; Yalabik et al., 2013). Satisfied employees often have more positive emotions, attitudes, and evaluations of their work, leading to higher work engagement as a reciprocal response (Rayton and Yalabik, 2014; Wang et al., 2019).

Based on the relationships between trait mindfulness and work engagement, trait mindfulness and job satisfaction, job satisfaction and work engagement, the following hypothesis can be proposed:

*H2*: Job satisfaction mediates the relationship between trait mindfulness and work engagement among Chinese primary and secondary school teachers.

### 2.3 Perceived organizational support, work engagement, and job satisfaction

#### 2.3.1 Perceived organizational support

Perceived organizational support (POS) refers to employees’ perceptions of how much the organization values their contributions and cares about their well-being (Eisenberger et al., 1986; Rhoades and Eisenberger, 2002). Organizational support theory posits that when employees feel organizational concern for their work and lives, it strengthens their expectations and emotional interactions with the organization, fostering a sense of value and motivating them to achieve organizational goals (Eisenberger et al., 1986; Joo, 2010). According to social exchange theory, employees expect high levels of organizational support and reciprocate accordingly (Wayne et al., 2002). POS creates a sense of obligation to reciprocate (Akgunduz et al., 2018). It represents a positive interaction between the organization and employees. Organizational support reduces employees’ psychological distress under pressure (George et al., 1993) and fulfills their socio-emotional and well-being needs in the workplace (Eisenberger et al., 1986). Employees with high POS experience less burnout and job dissatisfaction (Eisenberger et al., 2001; Rhoades and Eisenberger, 2002). High organizational support significantly enhances the organization’s appeal to employees and reduces turnover (Ballinger et al., 2010).

Previous research indicates a positive correlation between POS and teacher well-being. POS helps teachers cope with work stress and protects their mental health (Kurtessis et al., 2017; Runhaar et al., 2013). Studies on secondary school teachers show that when they perceive organizational support, they strive to achieve teaching goals as a form of reciprocation (Bai et al., 2022).

#### 2.3.2 Perceived organizational support and work engagement

Perceived organizational support enhances employee work engagement (Alfes et al., 2013; Bakker and Demerouti, 2008; Imran et al., 2020). POS fosters work engagement by creating a supportive environment (Bakker and Demerouti, 2008; Eisenberger et al., 2001), cultivating a conducive atmosphere for engagement (Wang, 2023). Such supportive environments enhance employees’ sense of value and involvement (Bakker et al., 2011; Bakker and Demerouti, 2008; Eisenberger et al., 2001).

Perceived organizational support also plays a crucial role in boosting teacher work engagement. Studies have found that secondary school teachers’ POS predicts their work engagement (Shen and Zhang, 2017). Supportive organizational environments may mitigate the negative effects of work stress on teachers and strengthen their sense of engagement and value (Derakhshan et al., 2023).

Based on the relationships between trait mindfulness and work engagement, and POS and work engagement, the following hypothesis can be proposed:

*H3*: For Chinese primary and secondary school teachers, POS strengthens the positive effect of trait mindfulness on job satisfaction, with stronger moderation at higher POS levels.

#### 2.3.3 Perceived organizational support and job satisfaction

Extensive research shows that employees with high POS cope better with work stress, thereby enhancing job satisfaction (Cullen et al., 2014; Eisenberger et al., 2001; Rhoades and Eisenberger, 2002). Employees with high POS not only exhibit higher job satisfaction but also more positive work states and better outcomes (Appelbaum et al., 2019). Organizational support theory suggests that when organizations meet employees’ socio-emotional needs and recognize their efforts, employees experience higher job satisfaction (Eisenberger et al., 1986; Kurtessis et al., 2017; Rhoades and Eisenberger, 2002). According to social exchange theory and the principle of reciprocity, organizational understanding and support are reciprocated by employees through hard work, reducing turnover and improving performance (Rhoades and Eisenberger, 2002).

Evidence from teacher studies indicates that POS influences their job satisfaction, work conditions, and mental health (Wang, 2024).

Based on the relationships between trait mindfulness and job satisfaction, and POS and job satisfaction, the following hypothesis can be proposed:

*H4*: Among Chinese primary and secondary school teachers, POS amplifies the positive effect of trait mindfulness on work engagement, and this moderating effect intensifies as POS increases.

**Figure 1:**
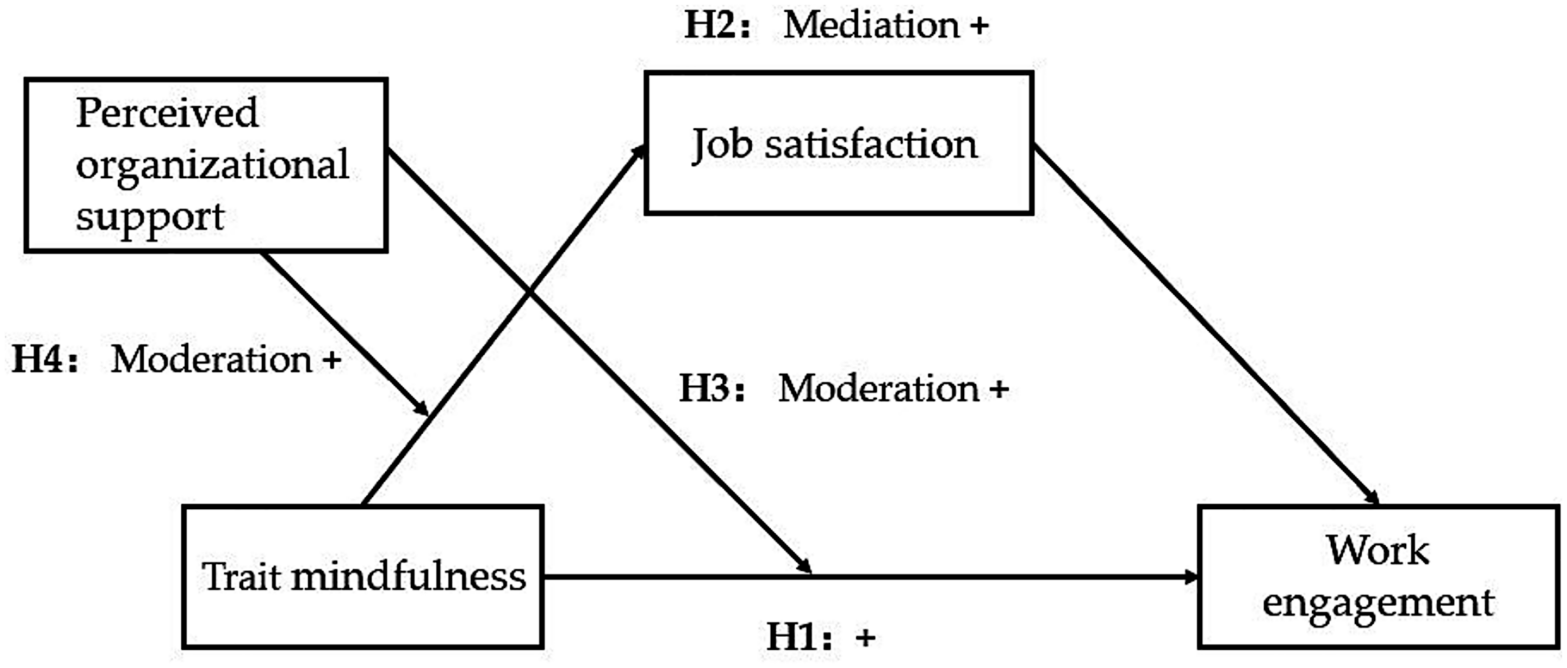
Hypothesized moderated mediation model.

To conclude, to explore the impact of trait mindfulness on work engagement among primary and secondary school teachers and its mechanisms, this study constructs a moderated mediation model (Figure 1). Specifically, trait mindfulness positively predicts work engagement, with job satisfaction as a mediator and perceived organizational support as a moderator.

To conclude, we propose our theoretical model in Figure 1.

## 3 Materials and methods

### 3.1 Participants

A convenience sampling method was used to survey 35 primary and secondary schools in inland provinces of China. These schools represented average local standards. A total of 3,225 teachers participated in the survey, including 1,202 male teachers (37.3%) and 2,023 female teachers (62.7%). Among them, 1,684 were primary school teachers (52.2%), 1,037 were junior high school teachers (32.2%), and 504 were senior high school teachers (15.6%). Teachers with less than 1 year of experience accounted for 182 (5.64%), those with over 6 years of experience accounted for 2,321 (71.97%), and those with over 20 years of experience accounted for 1,165 (36.12%). Urban teachers numbered 1,577 (48.9%), while rural teachers numbered 1,648 (51.1%).

The study was approved by the local educational science research department’s ethics committee. Before the survey, school principals were briefed on the research objectives, and their consent was obtained. Teachers were informed of the study’s purpose and their voluntary participation rights. All surveys were conducted online, and participants remained anonymous, with personal information strictly protected.

### 3.2 Measures

#### 3.2.1 Trait mindfulness scale

Trait mindfulness was measured using the Mindful Attention Awareness Scale (MAAS) developed by Brown and Ryan (2003), comprising 15 items. The scale has demonstrated good reliability and validity in Chinese populations (Black et al., 2012). It uses a 6-point Likert scale (1 = almost always; 6 = almost never), with higher scores indicating higher mindfulness levels. The fit indices from the confirmatory factor analysis of the scale indicated that *x^2^*/*df* = 21.774, CFI = 0.896, TLI = 0.905, RMSEA = 0.080, suggesting that the structural validity of the questionnaire is acceptable. The Cronbach’s *α* coefficient in this study was 0.93.

#### 3.2.2 Work engagement scale

Work engagement was assessed using the Utrecht Work Engagement Scale (UWES-9) developed by Schaufeli et al. (2006), consisting of 9 items. The scale’s reliability has been validated in multiple countries (Soininen et al., 2023). It includes three dimensions (vigor, dedication, and absorption) and uses a 7-point Likert scale (0 = never; 6 = always). Higher scores indicate higher work engagement. The fit indices from the confirmatory factor analysis of the scale indicated that *x^2^*/*df* = 37.290, CFI = 0.938, TLI = 0.907, RMSEA = 0.076, indicating that the structural validity of the questionnaire is acceptable. The Cronbach’s α coefficient in this study was 0.95.

#### 3.2.3 Job satisfaction scale

Job satisfaction was measured using the Teaching Satisfaction Scale (TSS) developed by Ho and Au (2006), comprising 5 items. It uses a 5-point Likert scale (1 = strongly disagree; 5 = strongly agree), with higher scores indicating higher job satisfaction. The fit indices from the confirmatory factor analysis of the scale indicated that *x^2^*/*df* = 72.303, CFI = 0.887, TLI = 0.913, RMSEA = 0.092, demonstrating that the structural validity of the questionnaire is acceptable. The Cronbach’s α coefficient in this study was 0.84.

#### 3.2.4 Perceived organizational support scale

Perceived organizational support was assessed using a scale developed by Ling et al. (2006). The scale has shown high reliability and validity in teacher-related studies (Liang et al., 2022; Wang and Tian, 2024). It uses a 6-point Likert scale (1 = strongly disagree; 6 = strongly agree), with higher scores indicating higher perceived organizational support. The fit indices from the confirmatory factor analysis of the scale indicated that *x^2^*/*df* = 11.641, CFI = 0.952, TLI = 0.946, RMSEA = 0.057, revealing that the structural validity of the questionnaire is acceptable. The Cronbach’s α coefficient in this study was 0.97.

### 3.3 Procedure

This study employs positivism as its theoretical framework to investigate causal mechanisms among variables via objective measurement and data analysis (Zhang and Zhang, 2023). A cross-sectional survey was conducted to examine core variable pathways. Methodologically, we adopted a quantitative paradigm using validated scales with documented reliability and validity. Data were collected through an online platform, distributed either directly by the authors or via teacher educators with close professional ties to teachers. After reviewing the study description, teachers voluntarily participated and completed the 5–10 min survey.

For data analysis, we first performed Harman’s single-factor test and confirmatory factor analysis (CFA) to evaluate common method bias. Subsequently, we tested the moderated mediation model using PROCESS Model 8 in SPSS to assess the mediating role of job satisfaction and the moderating role of perceived organizational support.

## 4 Results

### 4.1 Common method bias test

Since the data were collected through self-reporting, there may be a common methodological bias issue. To examine whether common method bias exists, the study employed two testing methods:

(1) Harman’s single-factor test was conducted, revealing five factors with eigenvalues greater than 1. The first factor accounted for 32.29% of the variance, which is below the critical threshold of 40%.
(2) Confirmatory factor analysis (CFA) was performed by setting the common factor to 1. The results showed poor model fit indices: *χ^2^/df* = 51.24, RMSEA = 0.125, CFI = 0.589, TLI = 0.571, SRMR = 0.131.

Therefore, the data do not exhibit significant common method bias.

### 4.2 Descriptive statistical analysis of variables

The means, standard deviations, and correlation matrix of the variables are presented in Table 1. Correlation analysis indicated significant positive relationships among the key variables. Specifically, trait mindfulness was significantly positively correlated with work engagement, job satisfaction, and perceived organizational support. There is a significant positive correlation between job satisfaction and work engagement. The sense of organizational support was positively correlated with job satisfaction and work engagement. These results provide support for further examination of the mediated role.

**Table 1:** Mean, standard deviation, and correlation coefficients of the main variables.

Variables	*M*(*SD*)	1	2	3	4
Trait mindfulness	4.14(0.77)	1			
Job satisfaction	3.832(0.59)	0.38^***^	1		
Work engagement	4.43(1.02)	0.44^***^	0.72^***^	1	
Perceived organizational support	3.57(0.96)	0.43^***^	0.52^***^	0.60^***^	1

***p < 0.001.

### 4.3 The relationship between trait mindfulness and work engagement: a moderated mediating model test

The SPSS macro program Model 8 was used to test the mediating effect of job satisfaction in the relationship between trait mindfulness and work engagement, as well as the moderating effect of perceived organizational support.

The moderated mediation analysis (Table 2) showed that trait mindfulness significantly and positively predicted job satisfaction (*β* = 0.17, *t* = 13.74, *p* < 0.001) and work engagement (*β* = 0.25, *t* = 14.04, *p* < 0.001). Job satisfaction also significantly and positively predicted work engagement (*β* = 0.79, *t* = 32.54, *p* < 0.001). The bootstrap 95% confidence interval for the mediating effect of job satisfaction did not include zero (Table 3), indicating that trait mindfulness directly predicted work engagement and indirectly predicted it through job satisfaction, suggesting a partial mediation model.

**Table 2:** Test of a moderated mediation model.

Variables	Job satisfaction				Work engagement			
	*β*	SE	t	95%CI	*β*	SE	*t*	95%CI
Trait mindfulness	0.15	0.02	7.71^***^	[0.11,0.19]	0.18	0.03	6.85^***^	[0.13,0.23]
Perceived organizational support	0.27	0.02	17.26^***^	[0.24,0.30]	0.29	0.02	12.66^***^	[0.24,0.33]
Job satisfaction					0.91	0.04	24.98^***^	[0.84,0.98]
Trait mindfulness × Perceived organizational support	0.04	0.02	2.93^*^	[0.01,0.07]	0.06	0.02	2.74^***^	[0.02,0.09]
*R* ^2^	0.30				0.60			
*F*	190.85^***^				489.42^***^			

^*^p < 0.001, ^***^p < 0.001.

**Table 3:** Conditional direct and indirect effect analysis.

Level of perceived organizational support	Conditional effect	Effect value	Bootstrap *SE*	Bootstrap LLCI	Bootstrap ULCI
Direct effect	*M* − 1*SD*	0.13	0.03	0.07	0.19
	*M* + 1*SD*	0.23	0.03	0.17	0.30
Indirect effect	*M*-1*SD*	0.10	0.02	0.05	0.15
	*M* + 1*SD*	0.18	0.02	0.13	0.22

**Figure 2:**
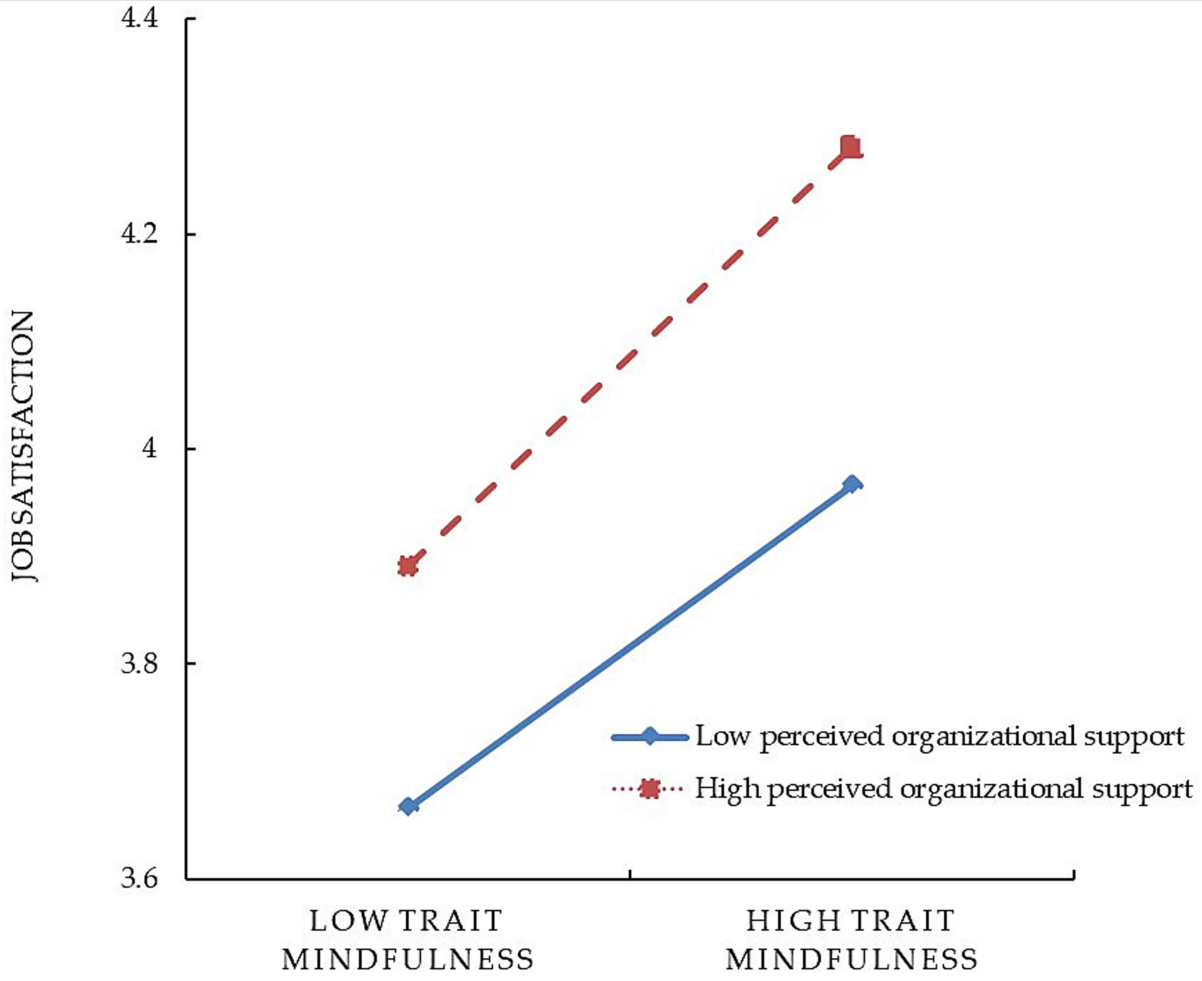
The moderating role of perceived organizational support between trait mindfulness and job satisfaction.

**Figure 3:**
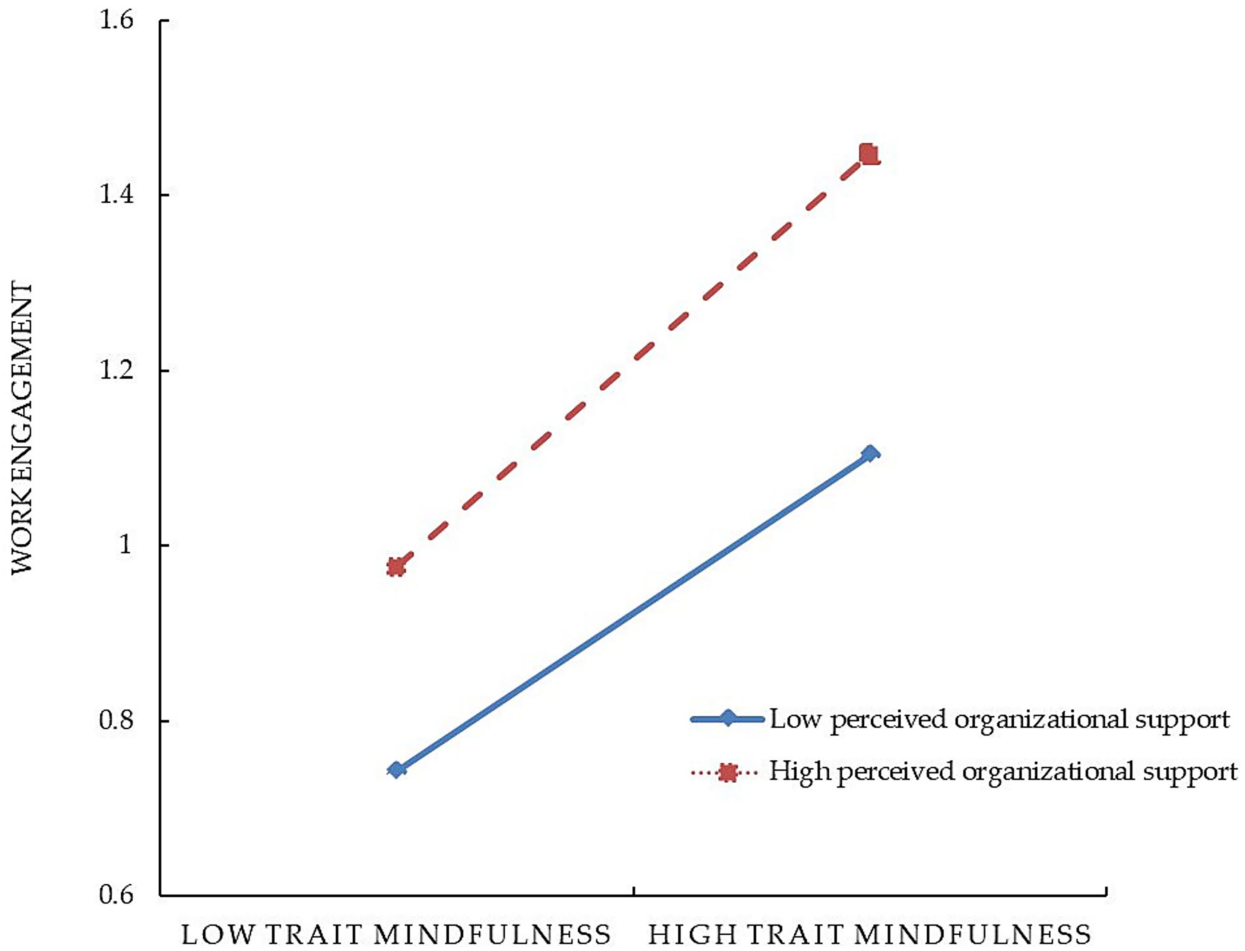
The moderating role of perceived organizational support between trait mindfulness and work engagement.

After incorporating perceived organizational support into the model, the interaction terms of trait mindfulness and perceived organizational support significantly and positively predicted work engagement (*β* = 0.05, *t* = 3.83, *p* < 0.001) and job satisfaction (*β* = 0.02, *t* = 2.38, *p* < 0.05). This indicates that perceived organizational support moderates both the direct relationship between trait mindfulness and work engagement and the relationship between trait mindfulness and job satisfaction. Simple slope analysis (Figures 2, 3) further illustrated the moderating role of perceived organizational support. Figure 2 shows that for individuals with a high perception of organizational support (*M* + 1*SD*), trait mindfulness positively predicts job satisfaction (simple slope = 0.19, *t* = 12.09, *p* < 0.001). In contrast, for those with low perceived organizational support (*M* − 1*SD*), trait mindfulness still significantly and positively influences job satisfaction (simple slope = 0.15, *t* = 10.42, *p* < 0.001), though the effect is weaker. This indicates that as an individual’s perception of organizational support increases, the impact of trait mindfulness on job satisfaction becomes stronger. From Figure 3, it can be observed that among individuals with low levels of organizational support (*M* − 1*SD*), trait mindfulness has a significant positive influence on work engagement (simple slope = 0.20, *t* = 9.99, *p* < 0.001). However, compared to those with high levels of organizational support (*M* + 1*SD*), the positive predictive effect of trait mindfulness on work engagement is even more pronounced (simple slope = 0.30, *t* = 13.13, *p* < 0.001). Therefore, it is evident that organizational support plays a significant moderating role in the relationship between trait mindfulness and work engagement.

## 5 Discussion

Based on positive psychology and previous research, the research constructed a moderated mediation model to clarify how trait mindfulness influences work engagement among primary and secondary school teachers through the mediating role of job satisfaction and the moderating role of perceived organizational support. The findings deepen the understanding of the mechanisms underlying teacher work engagement and offer practical insights for enhancing work engagement through trait mindfulness.

### 5.1 The relationship between trait mindfulness and work engagement

The results showed a significant direct effect of trait mindfulness on work engagement, providing new evidence for the relationship between teacher trait mindfulness and work engagement. Teachers with high trait mindfulness exhibited higher work engagement, consistent with studies on university teachers, preschool teachers, and other professions (Leroy et al., 2013; Li, 2022; Tao, 2022; Zhi and Wang, 2025). This study extended the findings to primary and secondary school teachers. The Conservation of Resources (COR) theory suggests that highly mindful teachers possess more psychological resources to mitigate stress, enabling them to work energetically and optimistically. Self-Determination Theory (SDT) posits that mindfulness helps teachers self-regulate and focus more on their work (Liu et al., 2020). Both perspectives explain the relationship between trait mindfulness and work engagement.

### 5.2 The mediating role of job satisfaction

Work engagement effectively enhances teacher well-being and performance. Mindfulness improves work engagement and job satisfaction by regulating attention and self-awareness. Exploring the mediating role of job satisfaction not only reveals the interaction between mindfulness and work engagement but also fills the research gap on the relationships among mindfulness, work engagement, and job satisfaction. This study found that trait mindfulness positively predicted work engagement through job satisfaction. Trait mindfulness helps teachers focus on their work, avoid negative distractions, and approach tasks with a calm mindset, reducing stress and anxiety, thereby increasing job satisfaction. Higher job satisfaction, in turn, promotes work engagement, as satisfied teachers evaluate their work more positively and reciprocate with higher engagement (Rayton and Yalabik, 2014; Wang et al., 2019). This study verified the positive relationship between trait mindfulness and job satisfaction, as well as the positive relationship between job satisfaction and work engagement. At the same time, the relationship between the three is included in the research, which confirms the mediating role of job satisfaction between trait mindfulness and work engagement, providing a new perspective on how trait mindfulness affects work engagement.

### 5.3 The moderating role of perceived organizational support

This study constructed a moderated mediation model based on perceived organizational support research, examining its role in the trait mindfulness-job satisfaction-work engagement pathway. The results showed that perceived organizational support moderated both the direct relationship between trait mindfulness and work engagement and the mediating pathway. Specifically, when perceived organizational support was high, trait mindfulness had a stronger predictive effect on work engagement and job satisfaction. Teachers with high perceived organizational support experienced fewer negative interferences. They also felt more valued by the organization, leading to higher job satisfaction and work engagement. These findings align with prior research (Alfes et al., 2013; Imran et al., 2020; Shen and Zhang, 2017). Incorporating perceived organizational support as a moderator enriches the understanding of the mechanisms linking trait mindfulness and work engagement.

## 6 Limitations and future research

The study has some limitations that need to be addressed in future research. First, the research adopts a cross-sectional design, which makes it difficult to accurately determine causal relationships between variables. Future research should employ longitudinal or experimental designs, such as cross-lagged panel designs, multilevel linear models, or manipulations of independent and mediating variables, to explore the relationship between trait mindfulness and work engagement. Second, the research relies solely on self-reported data, which may be subject to social desirability bias. Future research could incorporate multi-source reporting or expand data collection methods to enhance the objectivity of the results. In addition, the utilization of convenience sampling has the potential to give rise to limitations concerning applicability as well as other notable aspects. It is of great necessity that future research make conscious efforts to overcome these limitations.

## 7 Conclusion

The key conclusions are as follows: (1) Trait mindfulness significantly predicts work engagement among primary and secondary school teachers. (2) Job satisfaction mediates the relationship between trait mindfulness and work engagement. (3) Perceived organizational support moderates the relationship between trait mindfulness and work engagement. (4) Perceived organizational support moderates the relationship between trait mindfulness and job satisfaction, as well as the mediating effect of job satisfaction between trait mindfulness and work engagement.

In addition, schools can systematically construct a resource-enriched environment through implementing mindfulness training, the reinforcement of supportive policies, and the enhancement of job satisfaction to elevate teachers’ work engagement. Considering that trait mindfulness is characterized by relative stability and has a significant positive correlation with employees’ psychological resilience, work effort, job satisfaction, performance, etc. (Mesmer-Magnus et al., 2017), it is recommended to consider trait mindfulness as a reference element in teacher recruitment.

In summary, we believe these findings not only enrich theoretical research on teachers’ mindfulness and work engagement, also facilitate teachers in improving work performance and mental health, and in addition, provide practical insights for educational administrators.

## Data Availability

The raw data supporting the conclusions of this article will be made available by the authors, without undue reservation.
